# Artificial intelligence-powered coronary artery disease diagnosis from SPECT myocardial perfusion imaging: a comprehensive deep learning study

**DOI:** 10.1007/s00259-025-07145-x

**Published:** 2025-02-20

**Authors:** Ghasem Hajianfar, Omid Gharibi, Maziar Sabouri, Mobin Mohebi, Mehdi Amini, Mohammad Javad Yasemi, Mohammad Chehreghani, Mehdi Maghsudi, Zahra Mansouri, Mohammad Edalat-Javid, Setareh Valavi, Ahmad Bitarafan Rajabi, Yazdan Salimi, Hossein Arabi, Arman Rahmim, Isaac Shiri, Habib Zaidi

**Affiliations:** 1 Division of Nuclear Medicine and Molecular Imaging, Geneva University Hospital, Geneva 4, CH-1211 Switzerland; 2 Department of Physics and Astronomy, University of British Columbia, Vancouver, BC Canada; 3 Department of Integrative Oncology, BC Cancer Research Institute, Vancouver, BC Canada; 4 Institut de Biologie Valrose (IBV), Université Côte d’Azur, CNRS, Inserm, Nice, France; 5 Rajaie Cardiovascular Medical and Research Center, Iran University of Medical Sciences, Tehran, Iran; 6 Department of Cardiology, Bern University Hospital, Inselspital, University of Bern, Bern, Switzerland; 7 Department of Nuclear Medicine and Molecular Imaging, University of Groningen, University Medical Center Groningen, Groningen, Netherlands; 8 Department of Nuclear Medicine, University of Southern Denmark, Odense, Denmark; 9 University Research and Innovation Center, Óbuda University, Budapest, Hungary

**Keywords:** Coronary artery disease, Obstructive CAD, Deep learning, SPECT-MPI

## Abstract

**Background:**

Myocardial perfusion imaging (MPI) using single-photon emission computed tomography (SPECT) is a well-established modality for noninvasive diagnostic assessment of coronary artery disease (CAD). However, the time-consuming and experience-dependent visual interpretation of SPECT images remains a limitation in the clinic.

**Purpose:**

We aimed to develop advanced models to diagnose CAD using different supervised and semi-supervised deep learning (DL) algorithms and training strategies, including transfer learning and data augmentation, with SPECT-MPI and invasive coronary angiography (ICA) as standard of reference.

**Materials and methods:**

A total of 940 patients who underwent SPECT-MPI were enrolled (281 patients included ICA). Quantitative perfusion SPECT (QPS) was used to extract polar maps of rest and stress states. We defined two different tasks, including (1) Automated CAD diagnosis with expert reader (ER) assessment of SPECT-MPI as reference, and (2) CAD diagnosis from SPECT-MPI based on reference ICA reports. In task 2, we used 6 strategies for training DL models. We implemented 13 different DL models along with 4 input types with and without data augmentation (WAug and WoAug) to train, validate, and test the DL models (728 models). One hundred patients with ICA as standard of reference (the same patients in task 1) were used to evaluate models per vessel and per patient. Metrics, such as the area under the receiver operating characteristics curve (AUC), accuracy, sensitivity, specificity, precision, and balanced accuracy were reported. DeLong and pairwise Wilcoxon rank sum tests were respectively used to compare models and strategies after 1000 bootstraps on the test data for all models. We also compared the performance of our best DL model to ER’s diagnosis.

**Results:**

In task 1, DenseNet201 Late Fusion (AUC = 0.89) and ResNet152V2 Late Fusion (AUC = 0.83) models outperformed other models in per-vessel and per-patient analyses, respectively. In task 2, the best models for CAD prediction based on ICA were Strategy 3 (a combination of ER- and ICA-based diagnosis in train data), WoAug InceptionResNetV2 EarlyFusion (AUC = 0.71), and Strategy 5 (semi-supervised approach) WoAug ResNet152V2 EarlyFusion (AUC = 0.77) in per-vessel and per-patient analyses, respectively. Moreover, saliency maps showed that models could be helpful for focusing on relevant spots for decision making.

**Conclusion:**

Our study confirmed the potential of DL-based analysis of SPECT-MPI polar maps in CAD diagnosis. In the automation of ER-based diagnosis, models’ performance was promising showing accuracy close to expert-level analysis. It demonstrated that using different strategies of data combination, such as including those with and without ICA, along with different training methods, like semi-supervised learning, can increase the performance of DL models. The proposed DL models could be coupled with computer-aided diagnosis systems and be used as an assistant to nuclear medicine physicians to improve their diagnosis and reporting, but only in the LAD territory.

**Clinical trial number:**

Not applicable.

**Supplementary Information:**

The online version contains supplementary material available at 10.1007/s00259-025-07145-x.

## Introduction

Cardiovascular disease (CVD) is the leading cause of death in the US, with a morbidity rate of 48.6% among adults above 20 from 2017 to 2020 [1]. CVD mortality has increased sharply since 2010, and 41.2% of all CVD deaths were due to coronary artery disease (CAD) in 2020 [1]. CAD refers to a condition when lesions form atherosclerotic plaques in epicardial coronary arteries adversely affect the blood circulation in the heart [2, 3]. Invasive coronary angiography (ICA) is currently the gold standard for diagnosing CAD, and the diagnostic power of other methods is commonly evaluated against it [4]. Myocardial perfusion imaging (MPI) with single-photon emission computed tomography (SPECT) is a well-established tool enabling physicians to assess perfusion in the left ventricle (LV) non-invasively [5]. Variations in perfusion induced in the LV by CAD enables SPECT-MPI to diagnose and assess CAD. Meanwhile, visual interpretation of SPECT images is time-consuming and depends highly on the extent of the physician’s experience [6].

Computer-aided detection (CADe) and diagnosis (CADx) systems reduce the subjectivity of physician’s decision and interpretation time [7]. Such systems operate based on threshold values acquired from retrospectively collected groups of patients identified as normal or abnormal [7, 8]. Cedars Sinai’s quantitative perfusion SPECT (QPS) software is an example of such systems that employs the 17-segment polar map model proposed by American heart association (AHA) to quantify CAD severity by measuring the defect size and total perfusion deficit [9–11]. For best functionality, a specific standardized control database must be generated for various SPECT devices, radioisotopes, imaging procedures, etc., which poses a great limitation [8].

Recent advancements in artificial intelligence (AI) have broadened the applications of machine learning (ML) in cardiology and CADx systems [12–15]. In contrast to conventional ML techniques, deep learning (DL) methods do not require in-advance feature engineering and can learn directly from medical images once a sufficient training set is provided [7, 16]. The learning process occurs while an input, such as SPECT bull’s-eye polar map flows through various layers of a deep neural network, and numerous features are automatically extracted to classify images [16, 17]. Several studies have been directed into evaluating the accuracy of deep neural networks in CAD diagnosis in recent years, and different DL algorithms, such as convolutional neural networks (CNNs), have proven to result in promising outcomes [18–25].

In a study by Apostolopoulos et al. [22], InceptionV3 CNN was used along with random forest (RF) and other neural network classifiers to classify 566 CAD patients. They found that feeding InceptionV3 gives sub-optimal results compared to the expert reader’s (ER) accuracy, while the combination of InceptionV3 and RF can offer ER-equivalent results. However, their dataset was relatively small. In a larger study including 37,243 patients, Liu et al. [23], used the ResNet-34 algorithm without a dense layer to extract features from SPECT 2D circumferential count profile maps and then diagnosed perfusion abnormality by combining them with clinical features. They also compared the results of the DL approach with that of an automatic quantitative analysis software (Wackers-Liu CQ) based on defect size (DS) calculation. They reported that DL outperformed the software with a smaller variance (*p* < 0.01). However, their research lacked ICA as the standard ground truth for training and assessing the DL model.

Several studies used ICA as the standard ground truth [18–21]. Betancur et al. [18, 19] used multicentric datasets (1638 [18] and 1160 [19] patients) to predict CAD from SPECT-MPI. In both studies, they found that DL improved automatic CAD prediction. In another multicentric study, Otaki et al. [20] used explainable DL to detect obstructive CAD in a larger dataset (3578 patients) and to highlight diagnostically important spots for physicians. They reported that their DL model showed improved sensitivity with the same specificity as ERs’ visual and standard quantitative assessments. However, in [18] and [20] only stress SPECT-MPI images were used while rest images also bear useful information and can help train better models [26].

Collecting a large and balanced training dataset that represents the real-case population is challenging. Most of the patients with SPECT-MPI and ICA used for training a DL model are abnormal and this leads to biased estimations [27]. To reduce the inaccuracy of estimates for lower-risk patients, Miller et al. [21] included low-likelihood cases without ICA as normal patients in their dataset besides patients with ICA to improve the performance of their model. A solution to this challenge would be to include patients without ICA reports to the dataset and training models with unprecedented techniques, such as transfer learning or semi-supervised strategies.

In this study, we conducted comprehensive analysis to arrive at optimized DL-driven automated models for CAD diagnosis using various training strategies, including semi-supervised and transfer learning and inputting rest, stress, early, and late fusion polar maps into multiple DL algorithms. The present study incorporates patients diagnosed with CAD based on ICA or ERs’ review of SPECT-MPI images into the training procedure.

## Materials and methods

**Fig. 1:**
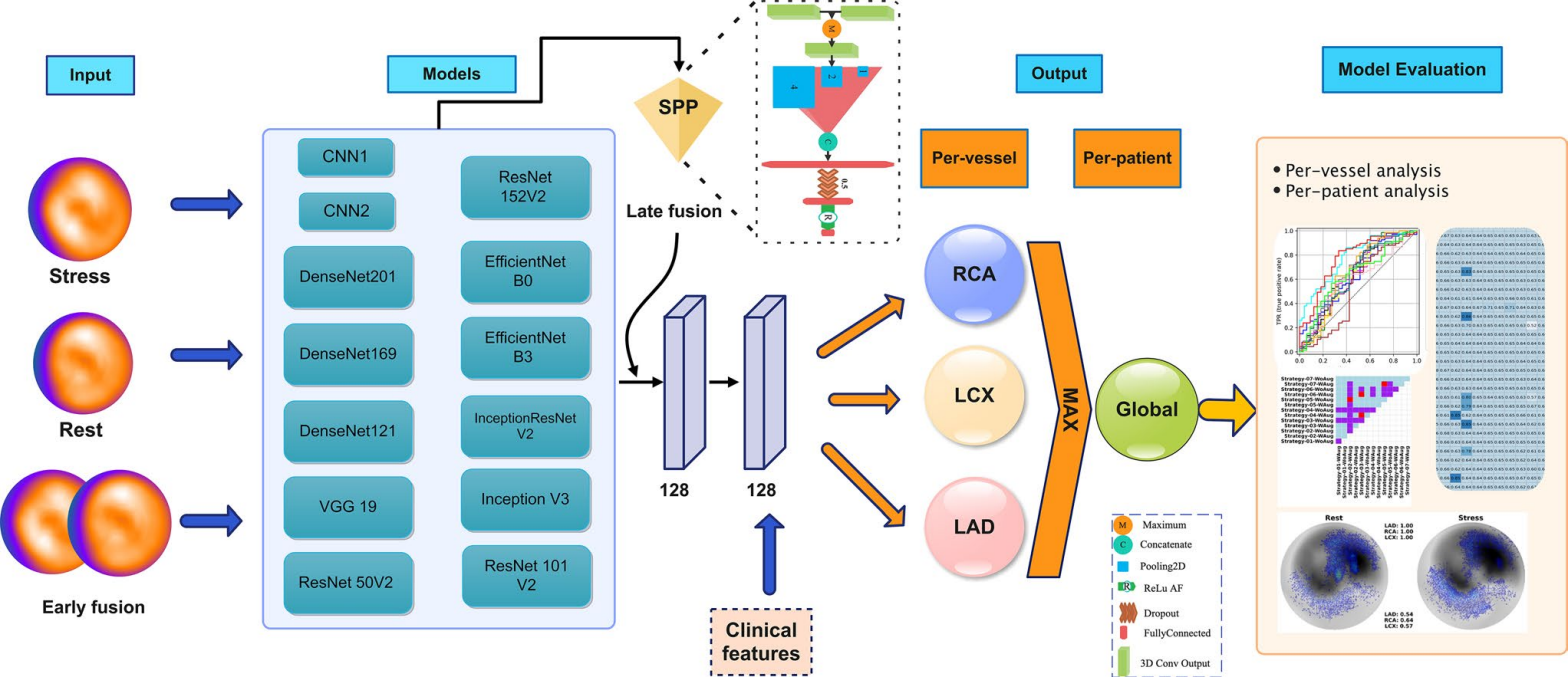
Descriptive illustration of the different steps taken in this study. SPP: spatial pyramid pooling 2D, RCA: right coronary artery, LCX: left circumflex artery, LAD: left anterior descending artery

Fig. 1 gives a general overview of the different steps involved in this study protocol

### Data collection

We included patients who underwent SPECT-MPI within 6 months before/after ICA from March 2019 to June 2021. SPECT-MPI was performed in one-day or two-day rest and stress protocol after weight-based administration of 555–740 MBq of ^99m^Tc-Sestamibi radiopharmaceutical. Perfusion images were acquired in a 180° orbit with automatic body contouring from 135° (RAO) to -45° (LPO) using a dual-head SPECT/CT system (Symbia™ T2, Siemens Healthcare). The energy peak was centered at 140 keV with a 15% (20 keV energy window width) symmetrical window. Matrix size and zoom factor were set to 64 × 64 and 1.45, respectively. Table 1 shows the characteristics of patients enrolled in this study. This study was performed in accordance with the principles of the Declaration of Helsinki. The Ethics Committee of Iran University of Medical Sciences granted approval (No. IR.IUMS.FMD.REC.1401.419).

### Image reconstruction and preprocessing

SPECT-MPI data were reconstructed using Cedars Sinai’s software [11] with ordered-subset expectation maximization (OSEM) algorithm. We used 4 iterations and 4 subsets for image reconstruction. The Butterworth filter with a 0.5 cutoff and order of 5 was applied to images. After reconstruction, QPS was used to extract polar maps of rest and stress states. Then, the extracted polar maps were converted to gray-scale images and normalized to the maximum value of each image. The retrieved polar maps had 290×290 matrix size, and no image resizing was applied to them.

### Ground truth definition

The enrolled dataset can be classified into two main cohorts based on the reference of CAD diagnosis, namely ICA and ERs’ diagnosis. For those with ICA reports, luminal diameter narrowing ≥ 50% in the left main artery or ≥ 70% in the left anterior descending artery (LAD), left circumflex artery (LCX), or right coronary artery (RCA) was defined as obstructive CAD [18–21].

For patients without ICA reports, two nuclear medicine specialists consensually made the final decision based on visual assessment of both non-attenuation-corrected (NAC) and attenuation-corrected (AC) perfusion images as standard clinical routine procedure for diagnosis. Assessments were made using QPS, quantitative gated SPECT (QGS), and other clinical features summarized in Table 1 based on a manual scoring system we refer to as Summed Scores (SS). SS is an ER-based correction to the American Heart Association (AHA)17-segment model.

In AHA 17-segment model [28], the severity of perfusion defects in each segment of a polar map is scored on a scale of 0 to 4 with scores reflecting 0 = normal, 1 = mild, 2 = moderate, 3 = severe, and 4 = absent perfusion. In this model, segments 1, 2, 7, 8, 13, 14, and 17 are related to LAD, segments 3, 4, 9, 10, 15 exhibit RCA function, and segments 5, 6, 11, 12, and 16 show whether LCX is healthy. While these assignments are not absolute, they are widely used in clinical and research settings to approximate coronary territories. The conventional Summed Stress Score (SSS) sums the scores of each segment in stress SPECT-MPI images. A region with SSS ≥ 4 is classified as abnormal, indicating significant perfusion defects and consequently vascular complications. Regions with scores below this threshold are classified as normal [28, 29].

In our SS model, nuclear medicine physicians followed the same approach as SSS, except that they manually edited the score of some segments according to each patient’s demographics where necessary. This was done to better reflect the combined diagnostic perspective incorporating all available clinical information for each patient. For patients who did not undergo ICA, our ERs focused on detecting the presence or absence of myocardial perfusion abnormalities by calculating SS for each coronary artery territory.

### Task and training strategy definition

In this study, we defined two main tasks for the models. In Task 1, we aimed to train DL models that can automatically classify patients into normal and abnormal categories based on NAC SPECT-MPI. Each image was labeled based on a consensus review of two nuclear medicine physicians at the same time. In Task 2, we aimed to predict ICA-based diagnosis from NAC SPECT-MPI. To train DL algorithms in this task, we used six different strategies of combining patients who only had ER-based diagnoses on SPECT-MPI data with those who had both ER- and ICA-based diagnoses. In this task, we attempted to use supervised, semi-supervised, and transfer learning methods. The following paragraph explains the data combination strategies in generating train, validation, and test sets.

First, a general primary set was considered as an initial set for each of the train, validation, and test sets including 141, 40, and 100 patients, respectively. These patients had both ICA reports and SPECT-MPI. In Strategies 1 and 4, we used the initial train set and trained DL models based on ICA reference. The difference between these two strategies is that the models in Strategy 4 are transfer learning from an ER-based diagnosis model (Task 1). In fact, the weights for each model in this strategy were obtained from those trained in Task 1. In Strategies 2, 5, and 6, we added 659 patients having only ERs’ diagnoses to the initial training set. The difference between these strategies is that in Strategy 2, training was performed based on both ICA and ERs, while in Strategies 5 and 6, we generated ICA labels for the added 659 patients and trained our models on all data based on ICA references. This is called pseudo-labeling. Also, the difference between Strategies 5 and 6 is that in Strategy 5, the DL models trained in Strategy 1 were used to infer labels in a semi-supervised manner, while in Strategy 6, the DL models trained in Strategy 4 were used for inference (transfer-semi-supervised learning). The validation and test sets were the same in all these strategies.

**Fig. 2:**
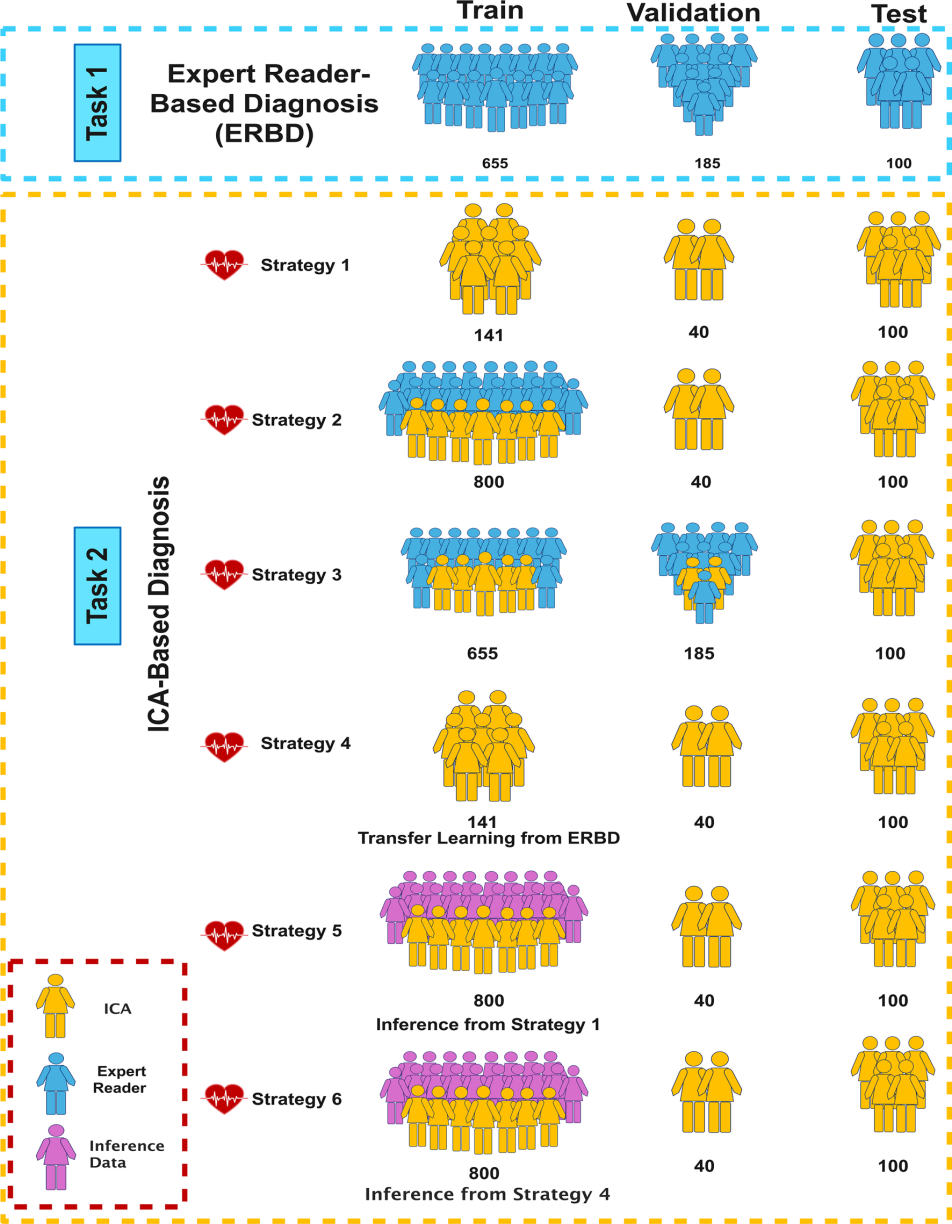
Illustration of the different tasks (ER-based and ICA-based diagnosis), and strategies used in this study to train, validate, and test the DL models for the prediction of CAD diagnosis from SPECT-MPI. ICA: invasive coronary angiography, ERBD: expert reader-based diagnosis

The only strategy with a different validation set is Strategy 3, in which we distributed the 659 patients without ICA to the initial train and validation sets, generating a train set of 655 patients and a validation set of 185 cases. Therefore, both ICA and ERs’ diagnoses were used for both training and validation. Fig. 2 shows an overview of the tasks and the corresponding strategies.

### Deep learning models

Data augmentation was performed on polar map images with ± 10^◦^ rotations. Each model in task 1 and task 2 (6 strategies) was used once with (WAug) and once without augmentation (WoAug). In this study, we implemented 13 different CNN models including CNN1, CNN2, DenseNet121, DenseNet169, DenseNet201, EfficientNetB0, EfficientNetB3, InceptionResNetV2, InceptionV3, ResNet101V2, ResNet152V2, ResNet50V2, and VGG19. CNN1 and CNN2 were inspired from Otaki [20] and Miller [21] studies, respectively.

We also compared the performance of models when fed with different inputs, including only rest polar maps, only stress polar maps, early fusion, and late fusion of polar maps. In early fusion, we fed stress and rest images at the same time as two-channel images. However, stress and rest were fed to the algorithms separately in late fusion, and 2D spatial pyramid pooling (SPP) was used in the final layer as in our previous study [30]. Prior to applying SPP, we used the maximum layer to get the single maximum matrix of the last output layer of stress and rest images.

Having 2 tasks, 6 different strategies in task 2, 13 DL algorithms, and 4 input types with and without data augmentation, we arrived at 728 different models. We used the same ending layer for all models inspired by Otaki’s study [20]. We used two consecutive 128 dense layers with an l2 regularizer of 0.01, followed by using the ReLU activation function and Dropout of 0.3 for each layer. We also concatenated clinical features to the last layer. These clinical features include age, gender, weight and height. For the output, we used a dense layer with 3 nodes and a sigmoid activation function. Adam optimizer and binary cross entropy were also implemented. The initial learning rate was set to 0.001, and ReduceLROnPlateau was used to reduce the learning rate by 0.1 if validation accuracy was not changed after 10 epochs. This process continued until the learning rate arrived at 1e − 7. The batch size and epoch number were set to 2 and 300, respectively.

### Model evaluation

We used the previously mentioned test set containing 100 randomly separated patients to evaluate our models. The patients in the test set were stratified by LAD, RCA, and LCX to ensure a similar rate of abnormal patients in the test set. All patients had both SPECT-MPI images for ER-based diagnosis and ICA reports. In Task 1, we evaluated the trained models against the ERs’ diagnosis, while in Task 2, models were assessed against the ICA ground truth. In both tasks, the performance of models was evaluated in per-vessel and per-patient approaches. Metrics, such as true negative (TN), true positive (TP), false negative (FN), false positive (FP), accuracy (ACC), area under the receiver operating characteristics curve (AUC), sensitivity (Sen), specificity (Spe), balanced accuracy (BAC), and precision (Pre) were then used to report models’ performance.

In the per-vessel approach, we concatenated probabilities of abnormal LAD, RCA, and LCX (≤ 100 each) and performed evaluation based on overall probability (≤ 300 total). However, in the per-patient approach, we considered the maximum probability of the 3 vessels, and estimation was performed per patient in the left ventricle over a probability of ≤ 100 [18, 19]. In this approach, a patient was labeled abnormal if one of each vessel met the previously mentioned inclusion criteria. Otherwise, it was considered normal.

The DeLong test was used to assess the significance level of the performance of each model by comparing receiver characteristic curves (ROCs). In addition, Wilcoxon rank sum test on bootstrap AUC was implemented to identify which training strategy, input, augmentation, and DL algorithm outperformed others. We applied these tests for both per-vessel and per-patient analyses. We used 0.05 as threshold for statistically significant differences.

Saliency maps were also created for the best model to show the model’s attention spots and explain the model’s decision [31]. Saliency maps are primarily intended to illustrate the regions of SPECT-MPI polar maps where the DL model is focusing to make predictions. These maps are not standalone diagnostic tools, but rather assistive visualizations designed to provide clinicians with insights into the model’s decision-making process. Ultimately, the final decision is derived from the probability outputs of the model, which are calculated based on all regions of the polar maps. Therefore, we provided an experienced nuclear medicine physician with both NAC and AC SPECT images, along with saliency maps and the probability of CAD occurrence in each artery predicted by our best model in per-patient analysis to compare the ER’s diagnosis with and without DL assistance. Finally, we compared the performance of our best DL model with an ER’s SS-based diagnosis.

All DL model development was performed in TensorFlow version 2.4 and evaluations and analyses were conducted in Python 3.9 and R 4.2.

## Results

**Table 1:** Characteristics of patients in both datasets

	Overall	ICA Train	ICA Test	ER
**Number of patients**	940 (100%)	181 (19.25%)	100 (10.64%)	659 (70.11%)
**Males**	709 (75.42%)	150 (82.87%)	87 (87.00%)	472 (71.62%)
**Age (mean ± SD)**	59.93 ± 11.57	59.89 ± 10.74	61.02 ± 10.93	59.77 ± 11.88
**Height (m)**	1.69 ± 0.11	1.70 ± 0.11	1.70 ± 0.09	1.68 ± 0.10
**Weight (kg)**	82.32 ± 15.63	82.35 ± 14.88	83.24 ± 14.47	82.17 ± 16.02
**BMI (kg/m** ^**2**^ **)**	28.82 ± 6.08	28.49 ± 5.77	28.80 ± 5.45	29.11 ± 6.18
**Chest pain**				
No pain	483 (51.38%)	87 (48.10%)	53 (53.00%)	343 (52.05%)
Typical	128 (13.62%)	31 (17.13%)	16 (16.00%)	81 (12.29%)
Atypical	277 (29.47%)	49 (27.07%)	25 (25.00%)	203 (30.80%)
Nonanginal	52 (5.53%)	14 (7.73%)	6 (6.00%)	32 (4.86%)
**DOE**	470 (50.00%)	97 (53.59%)	43 (43.00%)	330 (50.07%)
**Palpitation**	244 (25.96%)	46 (25.41%)	28 (28.00%)	170 (25.80%)
**CCU admission**	223 (23.72%)	35 (19.34%)	17 (17.00%)	171(25.95%)
**Diabetes Mellitus**	228 (24.25%)	49 (27.07%)	20 (20.00%)	159(24.13%)
**Hypertension**	553 (58.83%)	97 (53.59%)	56 (56.00%)	400(60.70%)
**Hypercholesterolemia**	460 (48.94%)	95 (52.49%)	43 (43.00%)	322(48.86%)
**Family history**	121 (12.87%)	28 (15.47%)	6 (6.00%)	87(13.20%)
**Stress**				
Exercise	559 (59.47%)	120 (66.30%)	63 (63.00%)	376 (57.10%)
Pharmacological	381 (40.53%)	61 (33.70%)	37 (36.00%)	283 (42.94%)
Dobutamine	7 (0.74%)	2 (1.10%)	0 (0.00%)	5 (0.76%)
Dipyridamole	374 (39.79%)	59 (32.60%)	37 (37.00%)	278 (42.18%)
**Disease type**				
1-vessel disease	248 (26.38%)	41 (22.65%)	23 (23.00%)	184 (27.92%)
LCX	77 (8.19%)	7 (3.87%)	4 (4.00%)	66 (10.01%)
RCA	40 (4.25%)	7 (3.87%)	4 (4.00%)	29 (4.40%)
LAD	131 (13.94%)	27 (14.91%)	15 (15.00%)	89 (13.51%)
2-vessel disease	122 (12.98%)	28 (15.47%)	16 (16.00%)	78 (11.84%)
LCX and RCA	32 (3.40%)	3 (1.66%)	2 (2.00%)	27 (4.10%)
LCX and LAD	42 (4.47%)	15 (8.29%)	9 (9.00%)	18 (2.73%)
RCA and LAD	48 (5.11%)	10 (5.52%)	5 (5.00%)	33 (5.01%)
3-vessel disease	102 (10.85%)	51 (28.18%)	28 (28.00%)	23 (3.49%) ​​
**Ground Truth**				
**Per-vessel**				
LCX (Normal, Abnormal)	687 (73.09%), 253 (26.91%)	105 (58.01%), 76 (41.99%)	57 (57.00%), 43 (43.00%)	525 (79.67%), 134 (20.33%)
RCA (Normal, Abnormal)	718 (76.38%), 222 (23.62%)	110 (60.77%), 71 (39.23%)	61 (61.00%), 39 (39.00%)	547 (83.00%), 112 (17.00%)
LAD (Normal, Abnormal)	617 (65.64%), 323 (34.36%)	78 (43.09%), 103 (56.91%)	43 (43.00%), 57 (57.00%)	496 (75.26%), 163 (24.73%)
**Per-patient**				
Normal, Abnormal	468 (49.79%), 472 (50.21%)	61 (33.74%), 120 (66.26%)	33 (33.00%), 67 (67.00%)	374 (56.75%), 285 (43.25%)

BMI: body mass index, DOE: dyspnea on exertion, CCU: coronary care unit, LCX: left circumflex artery, RCA: right coronary artery, LAD: left anterior descending artery, ICA: invasive coronary angiography, ER: expert reader

### Task 1: automation of ER-based diagnosis

The results of automated ER-based diagnosis (Task 1) are shown in Table 2 and Supplementary Figure S1. The results showed that WoAug DenseNet201 LateFusion (AUC = 0.89) and WoAug ResNet152V2 LateFusion (AUC = 0.83) outperformed other models in per-vessel and per-patient approaches, respectively. A comparison between the ROCs of these models is provided in supplementary Figure S2. Further performance metrics for each artery are shown in supplementary Table S1.

**Table 2:** The best-performing models in automated ER-based diagnosis in per-vessel and per-patient analyses

Approach	Augmentation	DL Network	Input	TP	TP	FN	FP	AUC	Acc	BAC	Sen	Spe	Pre
Per-vessel	Wo	DenseNet201	Late Fusion	181	53	15	51	0.89	0.78	0.78	0.78	0.78	0.51
	W	ResNet152V2	Early Fusion	175	54	14	57	0.85	0.76	0.77	0.79	0.75	0.49
Per-patient	Wo	ResNet152V2	Late Fusion	36	38	12	14	0.83	0.74	0.74	0.76	0.72	0.73
	W	ResNet152V2	Early Fusion	23	42	8	27	0.78	0.65	0.65	0.84	0.46	0.61

DL: deep learning, true negative: TN, true positive: TP, false negative: FN, false positive: FP, accuracy: ACC, area under the receiver operating characteristics curve: AUC, sensitivity: Sen, specificity: Spe, balanced accuracy: BAC, and precision: Pre

### Task 2: prediction of ICA-based diagnosis

The heat maps of AUCs of different models trained with different strategies in per-vessel and per-patient analysis are provided in supplementary Figure S3. Table 3 shows the two best-performing models for each training strategy in per-vessel and per-patient analyses. These models are selected not only based on high AUC but also on superior performance by all metrics. Further performance metrics for each artery are shown in supplementary Tables S2 and S3.

The best models for the prediction of ICA-based diagnosis were Strategy 3 WoAug InceptionResNetV2 EarlyFusion (AUC = 0.71) and Strategy 5 WoAug ResNet152V2 EarlyFusion (AUC = 0.77) in per-vessel and per-patient analyses, respectively. A comparison between ROCs of different final models is provided in supplementary Figure S4.

**Table 3:** The best-performing models in the prediction of ICA-based diagnosis in each training strategy in per-vessel and per-patient analyses

Approach	Strategy	Augmentation	DL Network	Input	TP	TP	FN	FP	AUC	Acc	BAC	Sen	Spe	Pre
Per-vessel	1	Wo	ResNet152V2	Rest	94	96	43	67	0.68	0.63	0.64	0.69	0.58	0.59
	1	W	InceptionV3	Stress	125	43	96	36	0.68	0.56	0.54	0.31	0.78	0.54
	2	Wo	InceptionResNetV2	Early Fusion	100	85	54	61	0.71	0.62	0.62	0.61	0.62	0.58
	2	W	CNN2	Late Fusion	110	73	66	51	0.69	0.61	0.60	0.53	0.68	0.59
	**3**	**Wo**	**InceptionResNetV2**	**Early Fusion**	**106**	**85**	**54**	**55**	**0.71**	**0.64**	**0.63**	**0.61**	**0.66**	**0.61**
	3	W	DenseNet201	Late Fusion	117	67	72	44	0.68	0.61	0.60	0.48	0.73	0.60
	4	Wo	ResNet101V2	Stress	106	82	57	55	0.67	0.63	0.62	0.59	0.66	0.60
	4	W	ResNet50V2	Stress	96	86	53	65	0.67	0.61	0.61	0.62	0.60	0.57
	5	Wo	CNN2	Early Fusion	83	99	40	78	0.68	0.61	0.61	0.71	0.52	0.56
	5	W	InceptionV3	Stress	122	44	95	39	0.66	0.55	0.54	0.32	0.76	0.53
	6	Wo	InceptionResNetV2	Late Fusion	107	79	60	54	0.67	0.62	0.62	0.57	0.66	0.59
	6	W	ResNet152V2	Early Fusion	98	85	54	63	0.67	0.61	0.61	0.61	0.61	0.57
Per-patient	1	Wo	CNN2	Early Fusion	16	55	12	17	0.70	0.71	0.65	0.82	0.48	0.76
	1	W	InceptionV3	Stress	25	31	36	8	0.67	0.56	0.61	0.46	0.76	0.79
	2	Wo	InceptionResNetV2	Early Fusion	16	55	12	17	0.70	0.71	0.65	0.82	0.48	0.76
	2	W	InceptionResNetV2	Early Fusion	22	40	27	11	0.69	0.62	0.63	0.60	0.67	0.78
	3	Wo	InceptionResNetV2	Early Fusion	20	49	18	13	0.71	0.69	0.67	0.73	0.61	0.79
	3	W	DenseNet201	Late Fusion	26	41	26	7	0.70	0.67	0.70	0.61	0.79	0.85
	4	Wo	DenseNet201	Early Fusion	16	52	15	17	0.69	0.68	0.63	0.78	0.48	0.75
	4	W	ResNet50V2	Stress	16	51	16	17	0.70	0.67	0.62	0.76	0.48	0.75
	**5**	**Wo**	**ResNet152V2**	**Early Fusion**	**20**	**56**	**11**	**13**	**0.77**	**0.76**	**0.72**	**0.84**	**0.61**	**0.81**
	5	W	VGG19	Late Fusion	15	57	10	18	0.66	0.72	0.65	0.85	0.45	0.76
	6	Wo	DenseNet201	Early Fusion	16	56	11	17	0.73	0.72	0.66	0.84	0.48	0.77
	6	W	ResNet50V2	Stress	18	54	13	15	0.71	0.72	0.68	0.81	0.55	0.78

DL: deep learning, true negative: TN, true positive: TP, false negative: FN, false positive: FP, accuracy: ACC, area under the receiver operating characteristics curve: AUC, sensitivity: Sen, specificity: Spe, balanced accuracy: BAC, and precision: Pre. The highlighted models in bold show the best performance in per-vessel and per-patient analyses

Table 4 shows the results of the DeLong test performed between the developed models for per-vessel and per-patient approaches to statistically compare the performance of the models. The ten top models are sorted and reported according to the number of models each one outperformed. It is noteworthy that this test is among the models for task 2 (ICA as ground truth). The best DL model in per-vessel analysis was Strategy 3 WoAug InceptionResNetV2 EarlyFusion performing significantly better than 572 models. In per-patient analysis, Strategy 5 WoAug_ResNet152V2_EarlyFusion was the best model tested based on ICA that outperformed the other 568 models.

**Table 4:** The results of the DeLong test performed on different models classifying patients based on per-vessel and per-patient approaches. The ten top models are sorted and reported according to the number of models each one outperformed

Analysis	Strategy	Augmentation	DL network	Input	Sum*
Per-vessel	3	Wo	InceptionResNetV2	Early Fusion	572
	2	Wo	InceptionResNetV2	Early Fusion	557
	5	Wo	InceptionResNetV2	Early Fusion	325
	5	Wo	CNN1	Rest	307
	1	Wo	CNN2	Early Fusion	271
	3	Wo	EfficientNetB3	Stress	199
	5	Wo	VGG19	Late Fusion	193
	1	Wo	DenseNet121	Early Fusion	189
	5	Wo	InceptionResNetV2	Rest	184
	3	Wo	CNN2	Late Fusion	155
Per-patient	5	Wo	ResNet152V2	Early Fusion	568
	5	Wo	CNN2	Early Fusion	368
	6	Wo	DenseNet201	Early Fusion	324
	5	Wo	ResNet152V2	Late Fusion	264
	5	Wo	InceptionV3	Stress	236
	1	Wo	ResNet101V2	Stress	202
	4	Wo	VGG19	Late Fusion	201
	5	Wo	CNN2	Late Fusion	195
	4	Wo	DenseNet121	Stress	176
	3	W	DenseNet201	Rest	169

Wo: without, W: with. *Sum of the number of models, each shown model outperforms the total 624 models

**Fig. 3:**
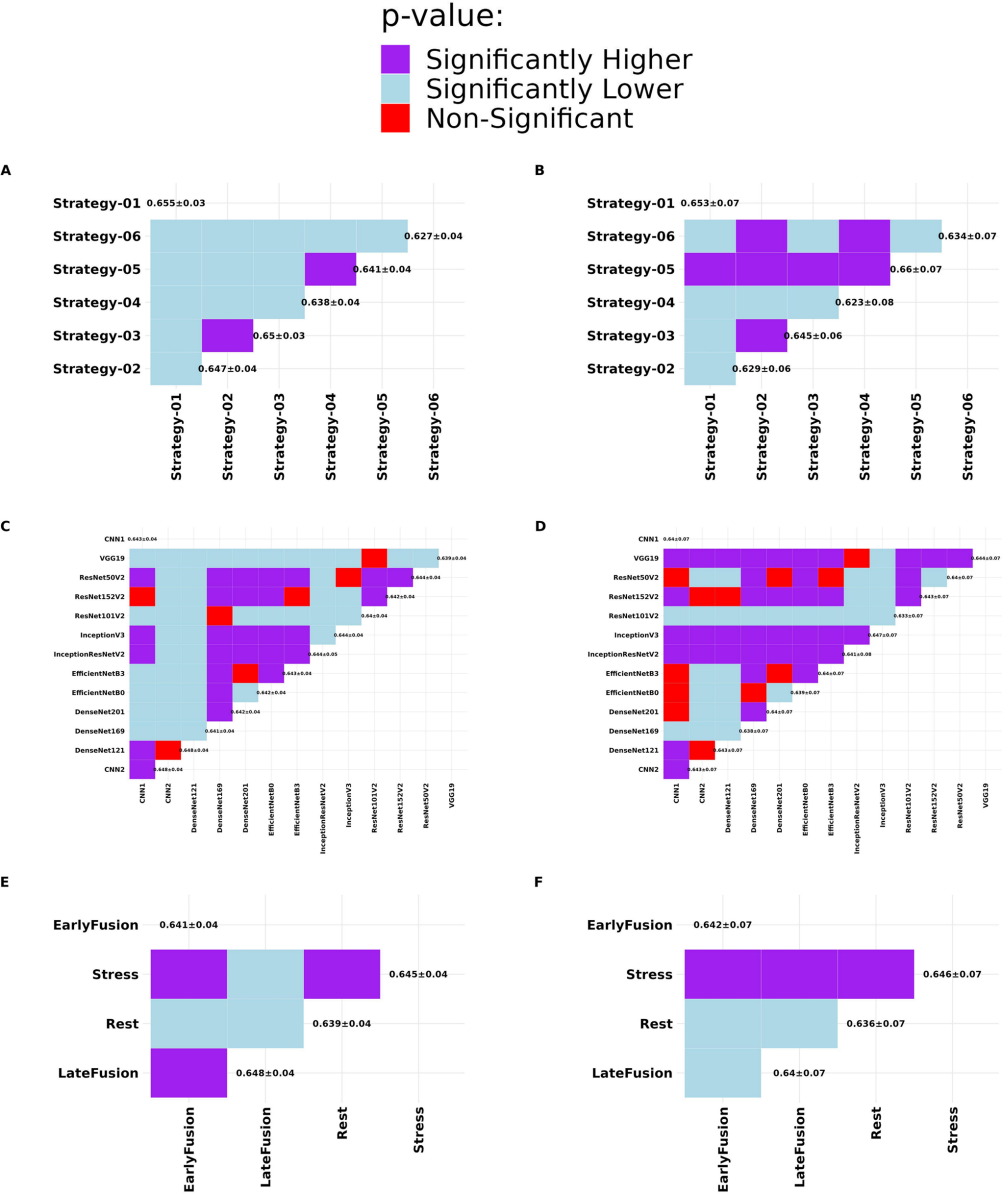
Wilcoxon rank sum test results comparing different training strategies (A and B), DL algorithms (C and D), and inputs (E and F) in per-vessel (left) and per-patient (right) methods of CAD prediction with ICA references (Task 2)

Moreover, to generally compare the outcome of different strategies, inputs, networks, and augmentation status, we performed a pairwise Wilcoxon rank sum test. To this end, we first calculated 1000 bootstraps on the test data for all models. Next, for example for the comparison of the strategies, we pooled the outcome of all models within each strategy together and applied pairwise Wilcoxon rank sum test between them. The results of this statistical test shown in Fig. 3 confirmed that Strategy 1, commonly DenseNet121 and CNN2, and LateFusion had the highest outcomes in per-vessel analysis, while Strategy 5, InceptionV3, and Stress input outperformed others in per-patient analysis. Performing augmentation resulted in superior performance in per-vessel analysis while not performing augmentation showed significantly higher performance in per-patient analysis.

**Fig. 4:**
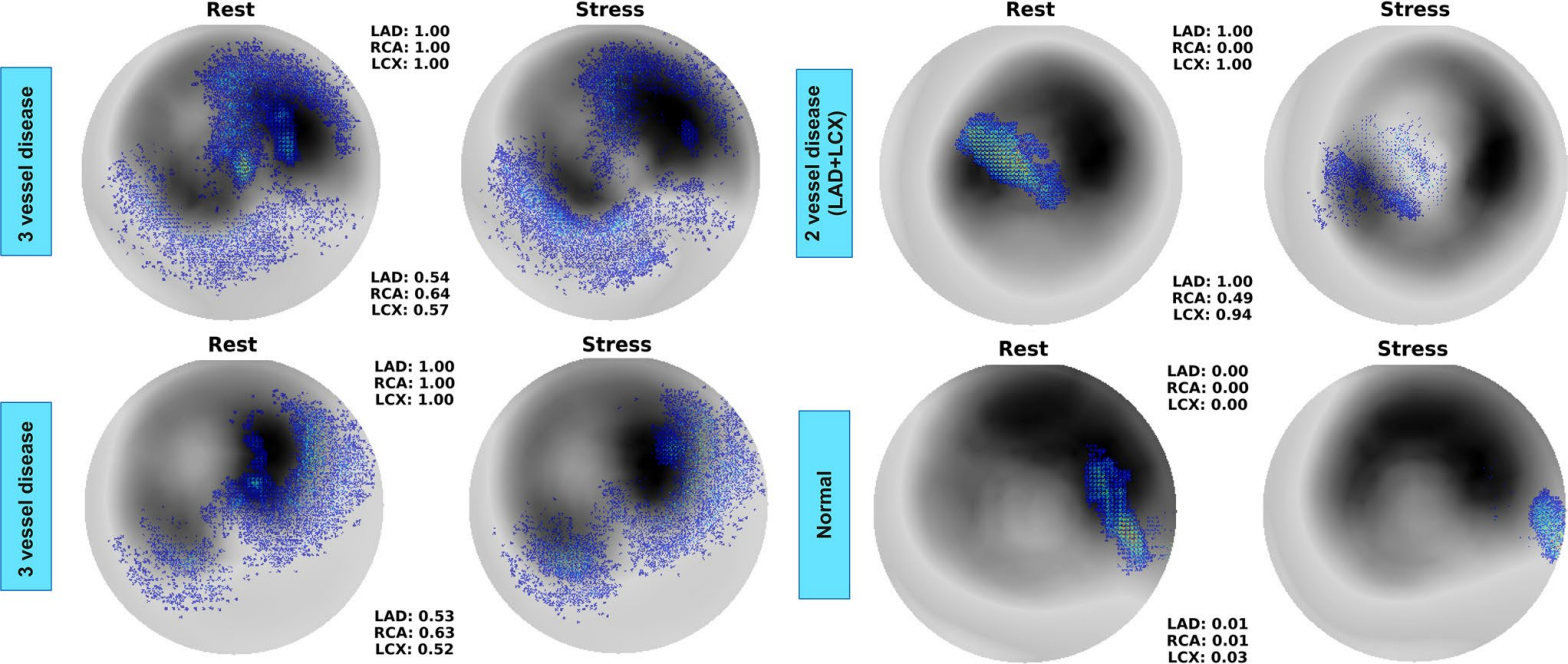
Saliency maps of 2 patients with 3- and 2-vessel diseases compared to a normal patient. LAD: left anterior descending artery, LCX: left circumflex artery, RCA: right coronary artery

Fig. 4 shows the saliency maps of 3 abnormal and 1 normal patient. As can be seen, saliency maps of three-vessel disease exhibit an overlap of features in rest and stress conditions. This is because the DL model might capture undetectable myocardial perfusion changes in both states when a patient is suffering from three-vessel CAD, even though the rest image does not show visible perfusion defects. Accordingly, the corresponding territories of each vessel can serve as suitable CAD indicators for the DL model. In the map provided for two-vessel CAD, the model is correctly not focused on all the territories, significantly concentrating on the LAD territory. This is while LCX is not highlighted although being predicted to be abnormal with a probability of 0.94. One should expect more focus also on LCX when it is highly predicted to be abnormal. In normal cases, some attention might still appear on LCX, but the assigned probabilities for all three territories remain near zero, reflecting the absence of significant abnormalities.

**Fig. 5:**
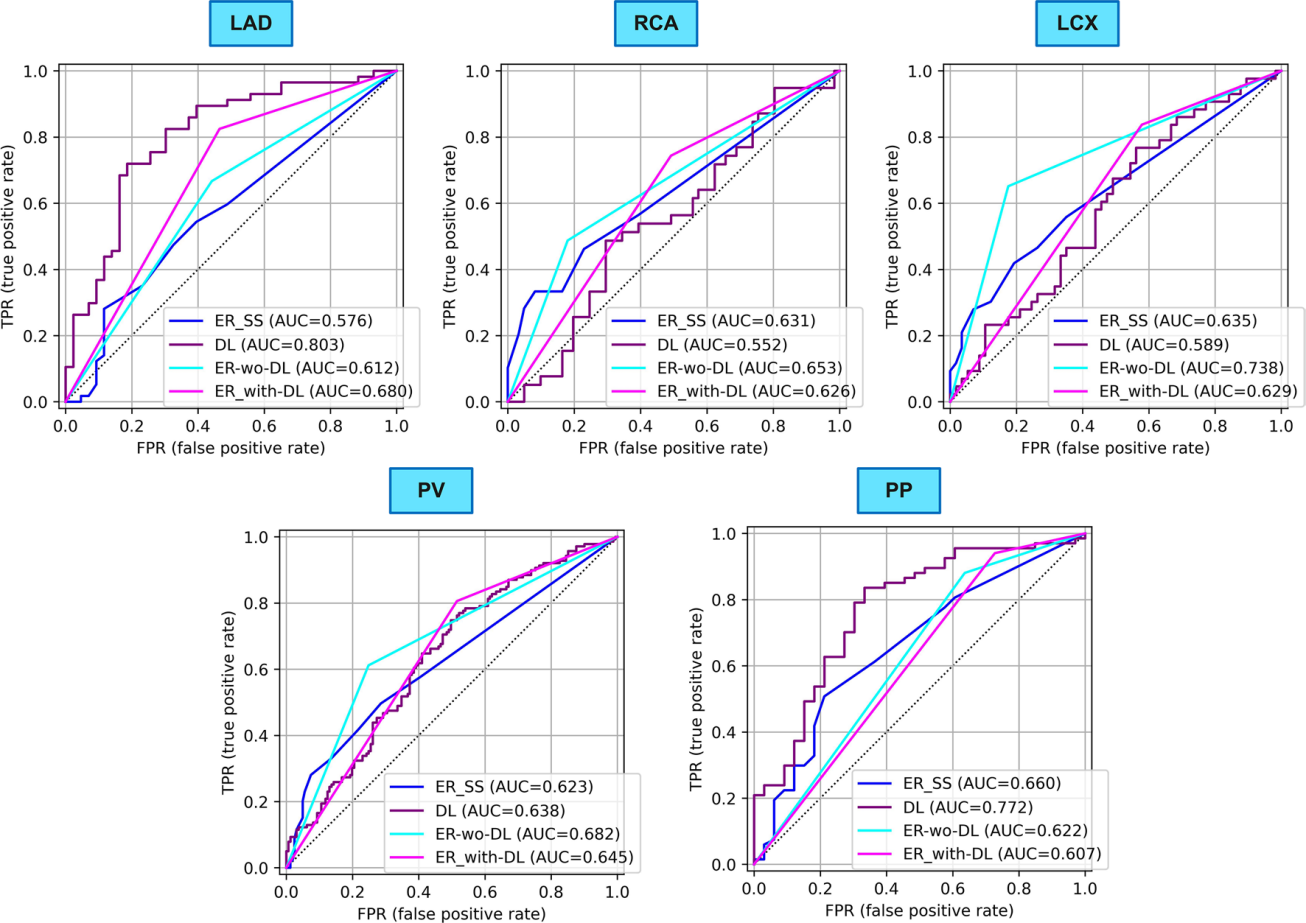
Comparison between the performance of our best DL model and ERs. LAD: left anterior descending artery, RCA: right coronary artery, LCX: left circumflex artery, PP: per-patient, PV: per-vessel, ER: expert reader, SS: summed score, DL: deep learning (Strategy-06-WoAug_ResNet152V2_EarlyFusion)

We also compared the performance of our best DL model in per-patient analysis in task 2 (Strategy 5 WoAug ResNet152V2 EarlyFusion) to SS-based ER’s diagnosis and to that of a new ER with and without DL assistance. Table 5; Fig. 5 show the superior performance of our DL model in the prediction of CAD in LAD (AUC = 0.80) compared to the SS-based diagnosis of the first (AUC = 0.58) and second (AUC = 0.61) ER. DL also improved the performance of the new ER to AUC = 0.68. The sensitivity and specificity of SS-based, DL, new ER without DL, and with DL were (0.35, 0.77), (0.61, 0.84), (0.67, 0.56), and (0.82, 0.53), respectively. The highest sensitivity (Sen = 0.82) and the highest specificity (Spe = 0.84) were achieved by ER with DL assistance and DL, respectively. SS-based diagnosis of CAD in LAD showed the lowest sensitivity (Sen = 0.35).

**Table 5:** Comparison between the performance of our best DL model and expert readers

	Metric	ER SS	ER without DL	DL	ER with DL
LAD	AUC	0.58	0.61	0.80	0.68
	Sen	0.35	0.67	0.61	0.82
	Spe	0.77	0.56	0.84	0.53
RCA	AUC	0.63	0.65	0.55	0.63
	Sen	0.33	0.49	0.54	0.74
	Spe	0.92	0.82	0.57	0.51
LCX	AUC	0.64	0.74	0.59	0.63
	Sen	0.30	0.65	0.67	0.84
	Spe	0.88	0.82	0.51	0.42
PP	AUC	0.66	0.62	0.77	0.61
	Sen	0.78	0.88	0.84	0.94
	Spe	0.42	0.36	0.61	0.27
PV	AUC	0.62	0.68	0.64	0.65
	Sen	0.33	0.61	0.61	0.81
	Spe	0.86	0.75	0.62	0.48

LAD: left anterior descending artery, RCA: right coronary artery, LCX: left circumflex artery, PP: per-patient, PV: per-vessel, ER: expert reader, SS: summed score, DL: deep learning (Strategy-06-WoAug_ResNet152V2_EarlyFusion)

In diagnosing CAD in RCA and LCX, the highest AUC was achieved by the new ER without DL assistance (AUC_RCA_ = 0.65, AUC_LCX_ = 0.74). Although DL did not perform well in RCA (AUC = 0.55), it enhanced the sensitivity of the new ER (Sen = 0.74) in RCA at the expense of reducing specificity (Spe = 0.51). In RCA, the lowest sensitivity (Sen = 0.33) and highest specificity (Spe = 0.92) were also achieved by SS-based diagnosis. The highest sensitivity (Sen = 0.84) in LCX was, however, achieved by the new ER with DL assistance.

In per-vessel analysis, the new ER achieved the highest performance (AUC = 0.68) without DL assistance. While SS-based diagnosis achieved the lowest sensitivity (Sen = 0.33), the new ER assisted with DL showed increasing sensitivity at the expense of decreasing specificity (Sen = 0.81, Spe = 0.48). In per-patient analysis, however, our DL model achieved the highest AUC and specificity (AUC = 0.77, Spe = 0.84) while the new ER with DL assistance showed the highest sensitivity but the lowest specificity (Sen = 0.94, Spe = 0.27). The performance of DL was balanced in terms of sensitivity and specificity.

**Table 6:** Comparison between previous studies and the present research with ICA and ER references

Ref.	Study	Patient Number and Input	Target	Model	Acc	Sen	Spe	AUC
ER-based diagnosis	Papandrianos et al. [32]	625 SA, HLA, VLA, S + R RGB	PP	RGB-CNN	0.93	-	-	0.94
	Zahiri et al. [33]	3,318 S + R PM RGB	PP	2D CNN	0.76	0.79	0.74	0.84
	Chen et al. [34]	979 SA	PP Tl	3D CNN	0.88	0.82	0.92	-
	Berkaya et al. [35]	192 SA, HLA, VLA, S + R RGB	PP	2D CNN	0.93	1.00	0.86	-
	Liu et al. [23]	37,243 S Recon profile	PP	2D CNN	0.83	0.74	0.85	0.87
	Magboo et al. [24]	192 SA, HLA, VLA, S + R RGB	PP	2D CNN	0.84	1.00	-	-
	This Study	940 S + R PM	PP PV	ResNet152V2 DenseNet201	0.74 0.78	0.76 0.78	0.72 0.78	0.83 0.89
ICA-based diagnosis	Apostolopoulos et al. [36]	216 S + R AC/NAC PM	PP	VGG16	0.75	0.75	0.73	-
	Apostolopoulos et al. [22]	556 S + R AC/NAC PM + Clinical	PP	InceptionV3 + RF	0.78	0.77	0.79	0.79
	Betancur et al. [18]	1,638 Raw + TPD PM	PP PV	CNN	- -	0.82 0.70	- -	0.80 0.76
	Betancur et al. [19]	1,160 Sup + SU TPD PM	PP PV	CNN	- -	- -	- -	0.81 0.77
	Otaki et al. [20]	3,578 S PM Perf Mot Thic	PP PV	2D CNN 10 CV	- -	0.61 -	0.84 -	0.83 0.79
	Miller et al. [37]	240 (Test)		Otaki	-	-	-	0.79
	Miller et al. [21]	828 + 511 S SU PM	PP	2D CNN	-	0.88	0.84	0.93
	Papandrianos et al. [25]	224 SA, HLA, VLA, S + R RGB	PP	RGB-CNN	0.94	0.94	0.78	0.93
	This Study	940 S + R PM	PP PV	ResNet152V2 InceptionResNetV2	0.76 0.64	0.84 0.61	0.61 0.66	0.77 0.71

ICA: invasive coronary angiography, ER: expert reader, PP: per-patient, PV: per-vessel, S: stress, R: rest, RGB: red green blue, AC: attenuation-corrected, NAC: non-AC, PM: polar map, TPD: total perfusion deficit, Sup: supine, SU: semi-upright, Perf: perfusion, Mot: motion, Thic: thickening, SA: short axis, HLA: horizontal long axis, VLA: vertical long axis

## Discussion

In this study, we developed DL models to automatically diagnose CAD using NAC SPECT-MPI with ERs’ diagnosis as reference (Task 1). We also trained models that can use NAC SPECT-MPI data to predict ICA-based diagnosis (Task 2). The different variables used to construct models in this study included the train/validation/test strategy (Fig. 2), the input of the models (rest, stress, early-, and late-fusion rest/stress polar map images), the deep network utilized, and whether data were augmented or not. In Task 1, models were trained and evaluated using the ERs’ diagnosis. However, in Task 2, we implemented semi-supervised and transfer learning along with 6 different combinations of training data. In addition, models were evaluated against the ICA ground truth in this task. The models’ performance was evaluated in terms of per-vessel and per-patient analyses in both tasks.

Table 6 compares previous studies with our study in terms of ER-based and ICA-based diagnosis. The results of Task 1 show that in automatic ER-based diagnosis, ResNet152V2_LateFusion fed with not-augmented data outperformed other models in per-patient analysis (AUC = 0.83). Several studies used only ERs’ diagnoses as references and analyzed the results using the per-patient approach [23, 33, 34]. Zahiri et al. [33] used 3,318 stress and rest polar maps and achieved an AUC of 0.84. In another study by Liu et al. [23], 37,243 stress 2D circumferential count profiles were used to achieve an AUC of 0.87. Chen et al. [34] used a 3D CNN model to classify 979 short-axis images, and the accuracy of their model was 0.88. What makes our study stand out is that we also analyzed models’ performance in a per-vessel approach.

In the per-vessel analysis of models developed in Task 1, our ResNet152V2_LateFusion model also showed acceptable performance with an AUC of 0.80. However, DenseNet201_LateFusion was the best-performing model achieving an AUC of 0.89. In a study using per-vessel analysis to evaluate the extent of agreement between their model and ER’s diagnosis, Spier et al. [38] used Graph CNN to classify normal and abnormal patients and localize CAD in MPI polar maps using 17- and 3-segment divisions. They achieved acceptable results for their model with an agreement of 0.79, sensitivity of 0.83, and specificity of 0.71. It is obvious that our DenseNet201_LateFusion model performed better. It is worth noting that this model showed a promising performance also in per-patient analysis.

In Task 2, we were inspired by a study by Miller et al. [21] in which they used normal patients with a low likelihood of CAD, along with patients with ICA reports for data augmentation and enhanced CAD prediction. Thus, we used SPECT-MPI polar maps with ER-based diagnosis along with patients with ICA-based diagnosis and tested several strategies of data combination, transfer learning, and semi-supervised learning to train DL models. In this task, we compared the DL models in two ways to determine the best-performing models in the prediction of ICA-based diagnosis regarding global and mean performance in each per-vessel and per-patient analyses. The results indicate that Strategy 3 WoAug InceptionResNetV2 EarlyFusion proved to be the best-performing model globally in per-vessel analysis. This model was trained and validated on a combined dataset of patients with ER- and ICA-based diagnoses as ground truth. However, Wilcoxon rank sum test results comparing variables in terms of mean AUC showed that using Strategy 1 of data combination, DenseNet121 or CNN2 as the network, and late fusion as input, provides the optimum combination of variables in average. Strategy 1 includes preparing a dataset of patients who have undergone both ICA (to be used as ground truth) and SPECT-MPI (to be used as input). However, collecting such datasets is challenging.

In per-patient analysis, Strategy 5 WoAug ResNet152V2 EarlyFusion model outperformed other models. In this model, patients without ICA were first inferenced in a semi-supervised manner based on models trained in strategy 1. Then, they were used to train and validate the models along with the patients with ICA. However, statistical analysis demonstrated that Strategy 5 of data combination, InceptionV3 network, and stress polar maps inputs lead to the highest performance.

Although Strategy 1 of data combination led to best average performance in the per-vessel analysis, Strategy 5 overcame other data combinations in the per-patient approach. This highlights the promising results of our study in developing models with equivalent or superior performance to a method in which we only rely on a dataset with ICA as reference. This finding is aligned with the study by Miller et al. [21]. One strength of our study is that we also included normal and abnormal SPECT-MPI rest, stress, early, and late fusion polar maps based on ERs’ diagnosis without ICA. The reason to do so was to avoid bias in patient selection and to be able to evaluate all trained models on a test dataset that includes ICA reports. This contrasts with Miller et al. [21] where only patients with a low likelihood of CAD were used beside ICA. Our results also showed that fusing stress and rest polar maps and feeding them to DL algorithms as early or late fusion inputs instead of simple rest and stress images, enhances the performance of models. Such results could be implemented in future studies to improve outcomes.

Several studies have performed per-vessel and per-patient analyses using ICA reference. In two studies, Betancur et al. [18, 19] showed the potential of polar maps in predicting CAD. They achieved AUCs of 0.80 and 0.81 (per patient) and 0.76 and 0.77 (per vessel). In another study, Otaki et al. [20] used a large dataset (*n* = 3,587) to produce a generalizable DL model to predict CAD in per-vessel (AUC = 0.79) and per-patient (AUC = 0.83) analysis. Miller et al. [37] tested the model developed by Otaki et al. [20] on an external dataset and achieved an AUC of 0.79 in per-patient analysis. In another study, Papandrianos et al. [25] used reconstructed RGB images to predict CAD in 224 patients achieving an AUC of 0.93.

Our study achieved globally superior results in most models without-augmentation approaches, indicating that augmentation did not improve the performance. In contrast, the statistical tests showed that augmented inputs led to superior mean performance in per-vessel analysis. This is while in per-patient analysis, non-augmented data gave better mean results. This discrepancy may be due to the averaging among all models when comparing the mean and global performance. Performance may reduce in some combinations when averaging. Miller et al. [21] used the same augmentation method but applied on normal and no obstructive CAD patients. They reported that augmentation significantly improved performance. One of the reasons that our study showed opposing results is that we have 3 outputs, and each output belongs to a specific region. Rotated polar maps may confuse DL models. In addition, we applied the augmentation on all images and not only the normal ones.

Another novelty brought in this study is that we extended our investigation to compare the performance of our DL models in diagnosing CAD in the three main arteries of the LV based on SPECT-MPI. We also compared the performance of our best DL model to that of an ER. The results show that DL has the highest performance in CAD diagnosis in LAD and per-patient analysis. There is a balance between sensitivity and specificity in RCA, LCX, and per-patient analysis. However, ERs have high specificity but low sensitivity in SS-based CAD diagnosis in LAD, RCA, LCX, and per-vessel analysis. This is while the opposite is true for per-patient analysis. In other words, ERs have superior performance in finding normal segments in LAD, RCA, LCX, and per-vessel analysis, while they may miss a large group of abnormal ones. In per-patient analysis, however, the performance was relatively more balanced. Moreover, using DL-gained saliency maps and probabilities as assistive tools to help an ER showed that the model could improve sensitivity and decrease specificity. This result indicates that we can hypothesize that an ER can benefit from DL-gained probabilities and saliency maps in relating SPECT-MPI polar maps and ICA data, thus improving the sensitivity of the diagnosis compared to the SS-based approach. However, the relationships between saliency map abnormalities and specific angiographic parameters, such as stenosis severity, lesion location, and collateral circulation in ICA images, remains an open research question requiring further investigation.

This is where AI may play a crucial role in supporting physicians by suggesting potentially abnormal regions that might be overlooked. While such models may not always have perfect specificity, experts can review and correct for any false positives. As a result, combining the high sensitivity of AI with the specificity of experts may increase the overall diagnostic performance. However, this requires in-depth investigations comparing DL-assisted diagnosis of a greater number of nuclear medicine physicians who are trained and are adequately familiar with the performance of the models and how much they can trust them.

This study has some limitations. Our sample size with ICA reference was relatively small. To overcome this limitation, we used more SPECT-MPI polar maps with ER-based diagnosis. Our intention was to evaluate the diagnostic utility of combining ER- and ICA-based references to overcome the challenge of limited ICA data. While this approach may seem simplistic compared to a broader integration of heterogeneous data, it provides a foundation for future studies to build upon and explore more sophisticated models. In addition, we did not have an external validation set to validate our results. Another limitation is that we used ICA data acquired within ± 6 months of SPECT imaging. We also used a threshold of 70% narrowing for CAD diagnosis. Using SPECT data of nearer time ranges and fractional flow reserve (FFR) data for CAD diagnosis is recommended in future studies. Moreover, we did not use scatter- and attenuation-corrected SPECT-MPI images in model development. The motivation behind this choice was to avoid artifacts that may occur in the RCA and LCX territories following AC [39]. Additionally, this approach makes our model more applicable for centers that use standalone SPECT cameras. However, we recommend evaluating the effect of scatter and attenuation correction on DL models’ performance. In this study, we only used stress and rest polar maps as inputs to DL models. We recommend using also motion and thickening, 3D images, and 3D CNN. Moreover, a larger number of nuclear medicine physicians should be included in future research to separately investigate the benefit and potential role of DL-gained probabilities and saliency maps in improving ERs’ diagnostic performance.

## Conclusion

Our study confirmed the power of DL-based analysis of SPECT-MPI polar maps for diagnosing CAD. In the automation of ER diagnosis, models’ performance was promising showing accuracy close to expert-level analysis. It also showed that using different data combination strategies, such as integrating data with and without ICA references and utilizing training methods like semi-supervised learning, can enhance the performance of DL models in predicting ICA-based diagnosis. The proposed DL models could potentially be used as an assistant to nuclear medicine physicians as decision-support tools for identifying abnormalities in the LAD territory. However, developing a robust assistant for RCA and LCX requires further investigation.

## Electronic supplementary material

Below is the link to the electronic supplementary material.

Supplementary Material 1

## Data Availability

The data used in this work is not available.

## Code Availability

The trained model is available on GitHub at the following address: https://github.com/pinlab-group/DeepCardiac.
