# Supplementary material for: Artificial intelligence-powered coronary artery disease diagnosis from SPECT myocardial perfusion imaging: a comprehensive deep learning study
Source: Eur J Nucl Med Mol Imaging. 2025 Feb 20;52(8):3019–35. doi: 10.1007/s00259-025-07145-x (PMC12162751; doi:10.1007/s00259-025-07145-x)
Supplement: Supplementary file 1 — Supplementary Material 1 [file 259_2025_7145_MOESM1_ESM.pdf]

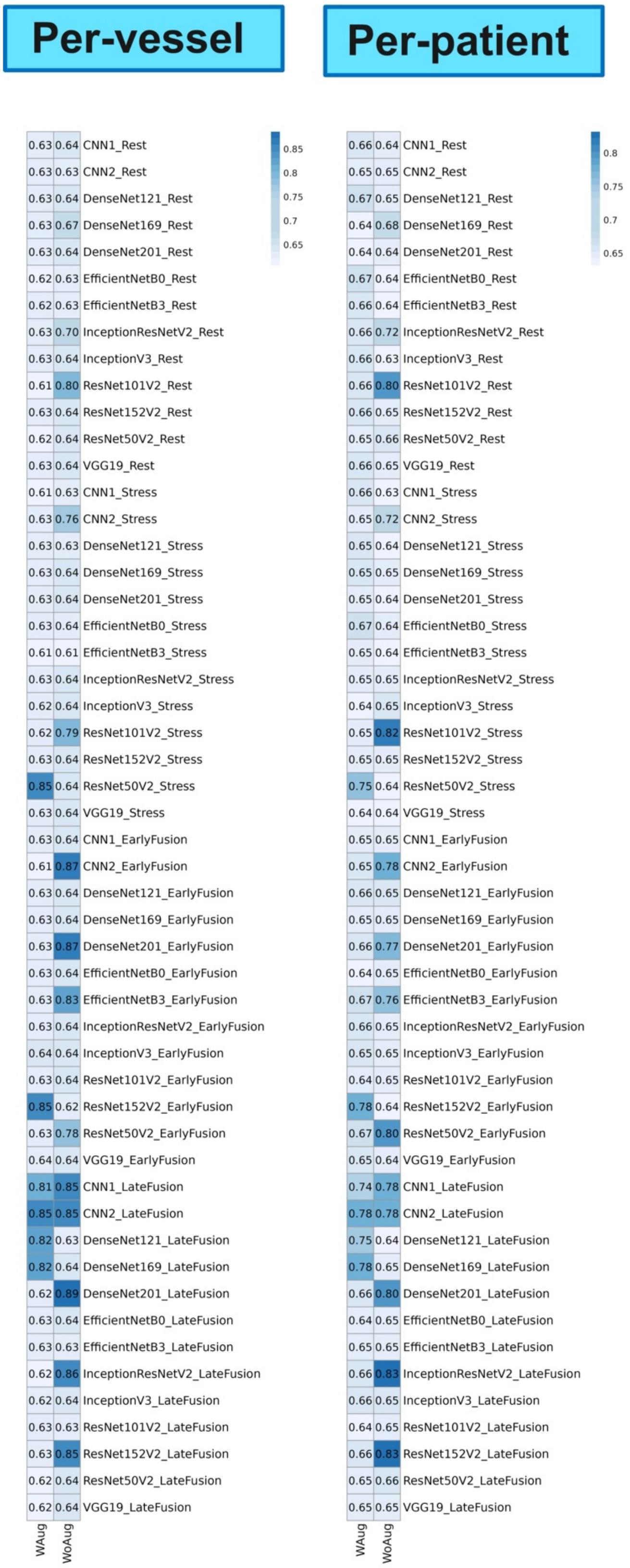

Figure S1. Heat maps of AUCs of different models in expert reader-based diagnosis strategy in per-vessel and per-patient analysis. Horizontal axis shows the with (WAug)/without (WoAug) data augmentation while vertical axis includes DL algorithms and inputs used.

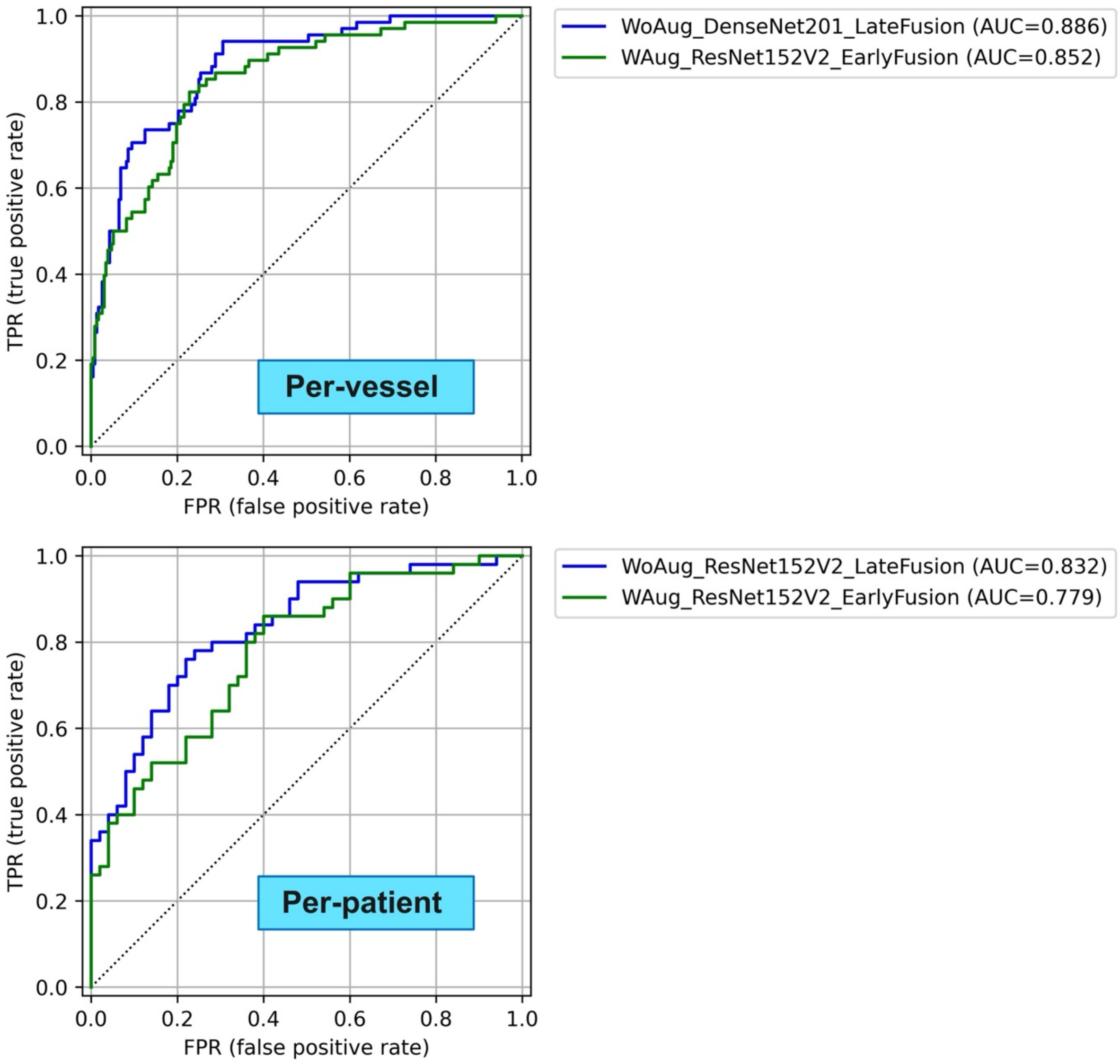

Figure S2. Comparison of best models in terms of Area Under ROC Curve (AUC) in expert reader-based diagnosis strategy in per-vessel and per-patients analysis. WoAug: Without Augmentation, WAug: With Augmentation.

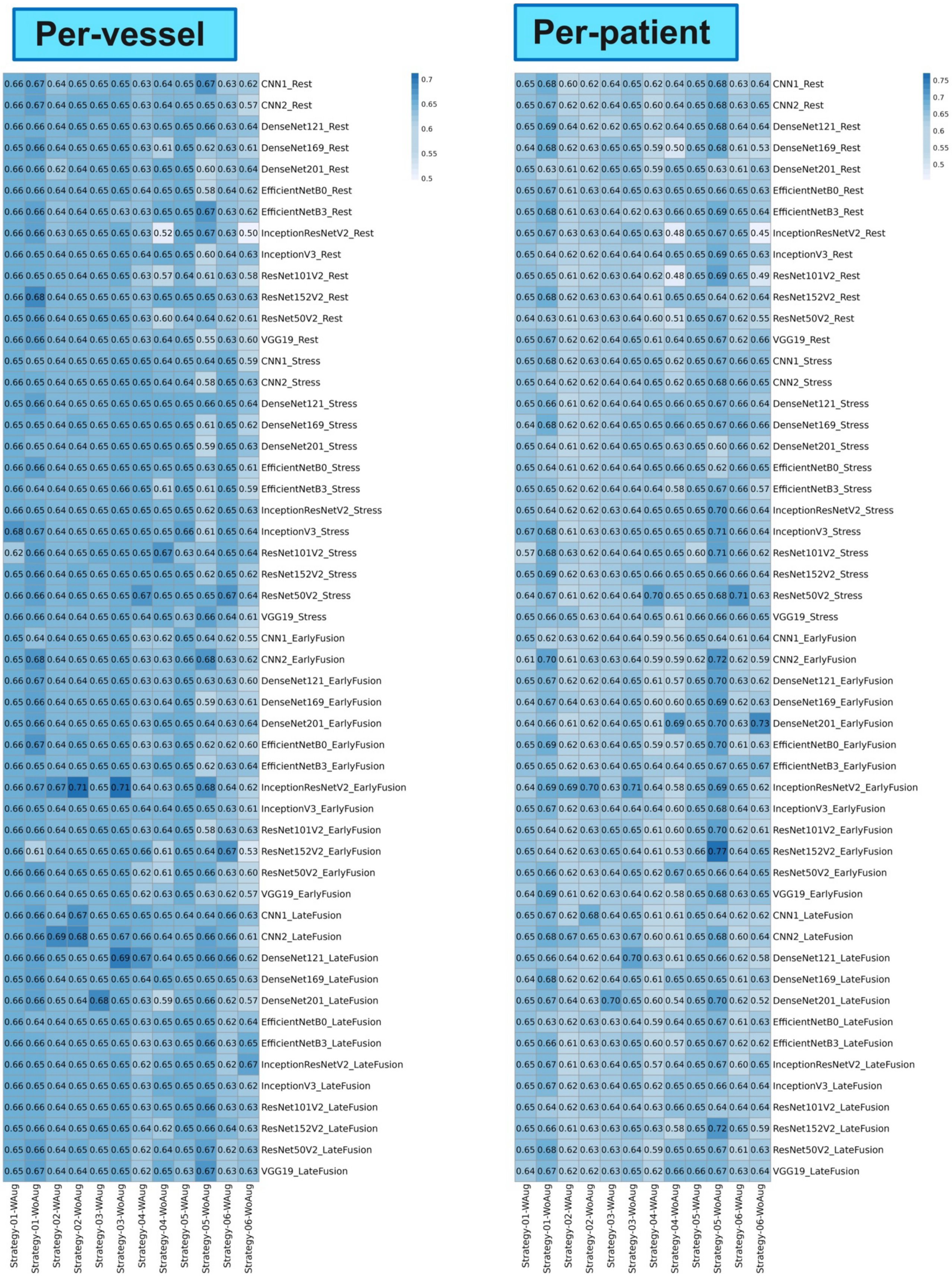

Figure S3. Heat maps of AUCs of different models and strategies in per-vessel and per-patient analysis in ICA-based diagnosis. Horizontal axis shows the 7 different training strategies with (WAug)/without (WoAug) data augmentation while vertical axis includes DL algorithms and inputs used.

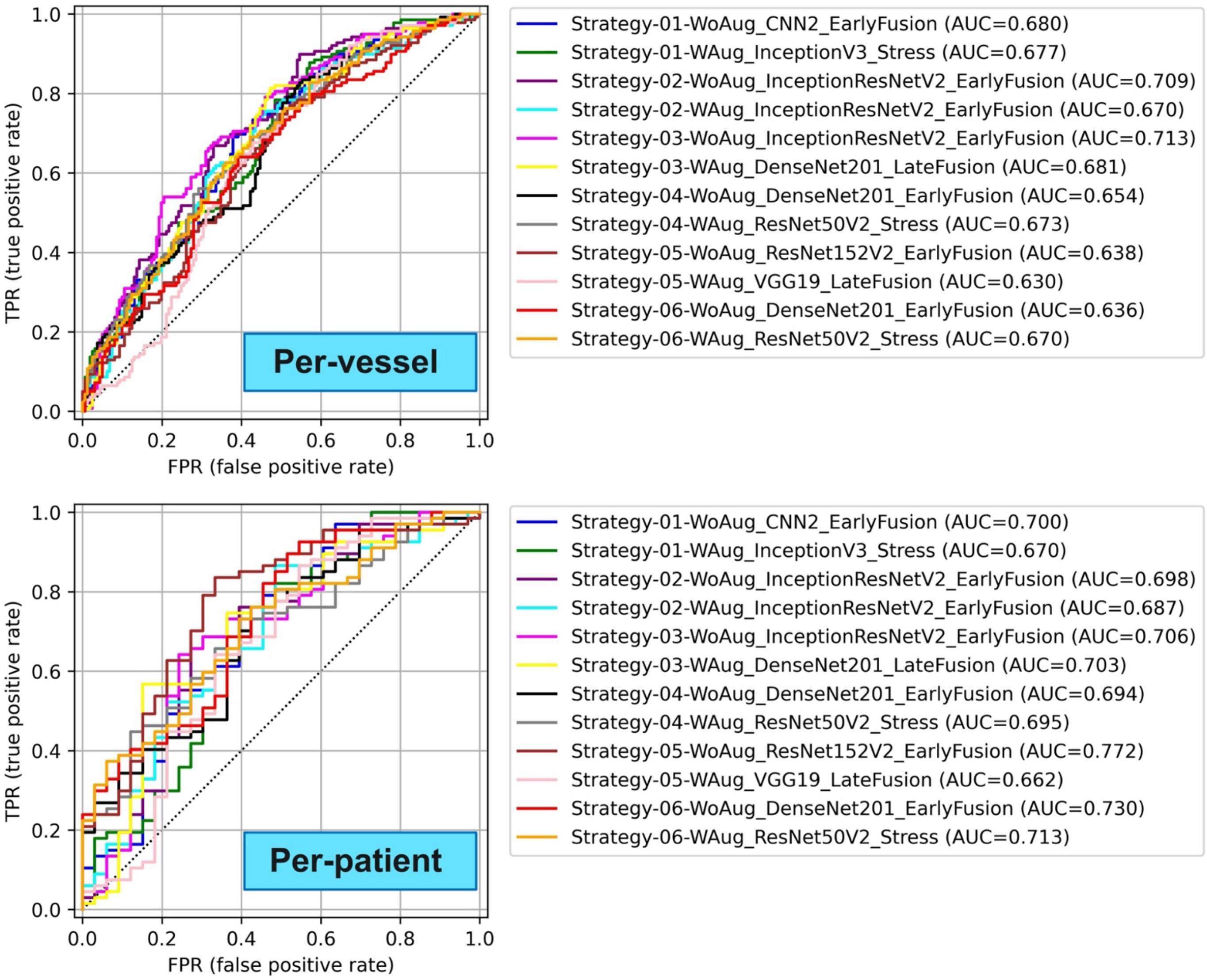

Figure S4. Comparison of best models in terms of Area Under ROC Curve (AUC) in each strategy in per-vessel and per-patients analysis in ICA-based diagnosis. WoAug: Without Augmentation, WAug: With Augmentation.

Table S1. The best model performance in expert reader-based diagnosis strategy in per-vessel and per-patient analysis.

**Analysis**	**Per-vessel**		**Per-patient**	
Strategy	WoAug	WAug	2-WoAug	2-WAug
Model	DenseNet201	ResNet152V2	ResNet152V2	ResNet152V2
Input	LateFusion	EarlyFusion	LateFusion	EarlyFusion
TN_LAD	55.00	54.00	59.00	54.00
TP_LAD	22.00	21.00	19.00	21.00
FN_LAD	8.00	9.00	11.00	9.00
FP_LAD	15.00	16.00	11.00	16.00
Acc_LAD	0.77	0.75	0.78	0.75
AUC_LAD	0.87	0.85	0.83	0.85
Sen_LAD	0.73	0.70	0.63	0.70
Spe_LAD	0.79	0.77	0.84	0.77
BAC_LAD	0.76	0.74	0.74	0.74
Pre_LAD	0.59	0.57	0.63	0.57
TN_RCA	60.00	68.00	60.00	68.00
TP_RCA	16.00	16.00	16.00	16.00
FN_RCA	2.00	2.00	2.00	2.00
FP_RCA	22.00	14.00	22.00	14.00
Acc_RCA	0.76	0.84	0.76	0.84
AUC_RCA	0.90	0.90	0.87	0.90
Sen_RCA	0.89	0.89	0.89	0.89
Spe_RCA	0.73	0.83	0.73	0.83
BAC_RCA	0.81	0.86	0.81	0.86
Pre_RCA	0.42	0.53	0.42	0.53
TN_LCX	66.00	53.00	58.00	53.00
TP_LCX	15.00	17.00	16.00	17.00
FN_LCX	5.00	3.00	4.00	3.00
FP_LCX	14.00	27.00	22.00	27.00
Acc_LCX	0.81	0.70	0.74	0.70
AUC_LCX	0.91	0.83	0.87	0.83
Sen_LCX	0.75	0.85	0.80	0.85
Spe_LCX	0.83	0.66	0.73	0.66
BAC_LCX	0.79	0.76	0.76	0.76
Pre_LCX	0.52	0.39	0.42	0.39
TN_PV	181.00	175.00	177.00	175.00
TP_PV	53.00	54.00	51.00	54.00
FN_PV	15.00	14.00	17.00	14.00
FP_PV	51.00	57.00	55.00	57.00
Acc_PV	0.78	0.76	0.76	0.76
AUC_PV	0.89	0.85	0.85	0.85
Sen_PV	0.78	0.79	0.75	0.79

Spe_PV	0.78	0.75	0.76	0.75
BAC_PV	0.78	0.77	0.76	0.77
Pre_PV	0.51	0.49	0.48	0.49
TN_PP	30.00	23.00	36.00	23.00
TP_PP	39.00	42.00	38.00	42.00
FN_PP	11.00	8.00	12.00	8.00
FP_PP	20.00	27.00	14.00	27.00
Acc_PP	0.69	0.65	0.74	0.65
AUC_PP	0.80	0.78	0.83	0.78
Sen_PP	0.78	0.84	0.76	0.84
Spe_PP	0.60	0.46	0.72	0.46
BAC_PP	0.69	0.65	0.74	0.65
Pre_PP	0.66	0.61	0.73	0.61

WoAug: Without Augmentation, WAug: With Augmentation, TN: True Negative, TP: True Positive, FN: False Negative, FP: False Positive, Acc: Accuracy, AUC: Area Under ROC Curve, Sen: Sensitivity, Spe: Specificity, BAC: Balanced Accuracy, Pre: Precision, LAD: Left Anterior Descending artery, RCA: Right Coronary Artery, LCX: Left Circumflex artery, PV: Per-vessel analysis, PP: Per-patient analysis.

Table S2. The best model performance in each strategy for per vessel analysis in ICA-based diagnosis.

Strategy	1-WoAug	1-WAug	2-WoAug	2-WAug	3-WoAug	3-WAug	4-WoAug	54-WAug	5-WoAug	5-WAug	6-WoAug	6-WAug
Model	ResNet152V2	InceptionV3	InceptionResNetV2	CNN2	InceptionResNetV2	DenseNet201	ResNet101V2	ResNet50V2	CNN2	InceptionV3	InceptionResNetV2	ResNet152V2
Input	Rest	Stress	EarlyFusion	LateFusion	EarlyFusion	LateFusion	Stress	Stress	EarlyFusion	Stress	LateFusion	EarlyFusion
TN_LAD	26.00	39.00	29.00	30.00	35.00	33.00	19.00	20.00	26.00	38.00	23.00	28.00
TP_LAD	35.00	12.00	30.00	26.00	26.00	23.00	41.00	36.00	38.00	14.00	33.00	35.00
FN_LAD	22.00	45.00	27.00	31.00	31.00	34.00	16.00	21.00	19.00	43.00	24.00	22.00
FP_LAD	17.00	4.00	14.00	13.00	8.00	10.00	24.00	23.00	17.00	5.00	20.00	15.00
Acc_LAD	0.61	0.51	0.59	0.56	0.61	0.56	0.60	0.56	0.64	0.52	0.56	0.63
AUC_LAD	0.65	0.63	0.67	0.64	0.66	0.65	0.66	0.59	0.66	0.63	0.60	0.62
Sen_LAD	0.61	0.21	0.53	0.46	0.46	0.40	0.72	0.63	0.67	0.25	0.58	0.61
Spe_LAD	0.60	0.91	0.67	0.70	0.81	0.77	0.44	0.47	0.60	0.88	0.53	0.65
BAC_LAD	0.61	0.56	0.60	0.58	0.64	0.59	0.58	0.55	0.64	0.56	0.56	0.63
Pre_LAD	0.67	0.75	0.68	0.67	0.76	0.70	0.63	0.61	0.69	0.74	0.62	0.70
TN_RCA	34.00	42.00	38.00	39.00	40.00	44.00	47.00	37.00	31.00	39.00	48.00	34.00
TP_RCA	30.00	18.00	22.00	23.00	26.00	21.00	18.00	26.00	27.00	19.00	19.00	22.00
FN_RCA	9.00	21.00	17.00	16.00	13.00	18.00	21.00	13.00	12.00	20.00	20.00	17.00
FP_RCA	27.00	19.00	23.00	22.00	21.00	17.00	14.00	24.00	30.00	22.00	13.00	27.00
Acc_RCA	0.64	0.60	0.60	0.62	0.66	0.65	0.65	0.63	0.58	0.58	0.67	0.56
AUC_RCA	0.66	0.66	0.65	0.65	0.70	0.66	0.64	0.67	0.62	0.64	0.68	0.64
Sen_RCA	0.77	0.46	0.56	0.59	0.67	0.54	0.46	0.67	0.69	0.49	0.49	0.56
Spe_RCA	0.56	0.69	0.62	0.64	0.66	0.72	0.77	0.61	0.51	0.64	0.79	0.56
BAC_RCA	0.66	0.58	0.59	0.61	0.66	0.63	0.62	0.64	0.60	0.56	0.64	0.56
Pre_RCA	0.53	0.49	0.49	0.51	0.55	0.55	0.56	0.52	0.47	0.46	0.59	0.45
TN_LCX	34.00	44.00	33.00	41.00	31.00	40.00	40.00	39.00	26.00	45.00	36.00	36.00
TP_LCX	31.00	13.00	33.00	24.00	33.00	23.00	23.00	24.00	34.00	11.00	27.00	28.00
FN_LCX	12.00	30.00	10.00	19.00	10.00	20.00	20.00	19.00	9.00	32.00	16.00	15.00
FP_LCX	23.00	13.00	24.00	16.00	26.00	17.00	17.00	18.00	31.00	12.00	21.00	21.00
Acc_LCX	0.65	0.57	0.66	0.65	0.64	0.63	0.63	0.63	0.60	0.56	0.63	0.64
AUC_LCX	0.67	0.68	0.77	0.72	0.73	0.68	0.66	0.72	0.71	0.67	0.67	0.71
Sen_LCX	0.72	0.30	0.77	0.56	0.77	0.53	0.53	0.56	0.79	0.26	0.63	0.65
Spe_LCX	0.60	0.77	0.58	0.72	0.54	0.70	0.70	0.68	0.46	0.79	0.63	0.63
BAC_LCX	0.66	0.54	0.67	0.64	0.66	0.62	0.62	0.62	0.62	0.52	0.63	0.64

Pre_LCX	0.57	0.50	0.58	0.60	0.56	0.58	0.58	0.57	0.52	0.48	0.56	0.57
TN_PV	94.00	125.00	100.00	110.00	106.00	117.00	106.00	96.00	83.00	122.00	107.00	98.00
TP_PV	96.00	43.00	85.00	73.00	85.00	67.00	82.00	86.00	99.00	44.00	79.00	85.00
FN_PV	43.00	96.00	54.00	66.00	54.00	72.00	57.00	53.00	40.00	95.00	60.00	54.00
FP_PV	67.00	36.00	61.00	51.00	55.00	44.00	55.00	65.00	78.00	39.00	54.00	63.00
Acc_PV	0.63	0.56	0.62	0.61	0.64	0.61	0.63	0.61	0.61	0.55	0.62	0.61
AUC_PV	0.68	0.68	0.71	0.69	0.71	0.68	0.67	0.67	0.68	0.66	0.67	0.67
Sen_PV	0.69	0.31	0.61	0.53	0.61	0.48	0.59	0.62	0.71	0.32	0.57	0.61
Spe_PV	0.58	0.78	0.62	0.68	0.66	0.73	0.66	0.60	0.52	0.76	0.66	0.61
BAC_PV	0.64	0.54	0.62	0.60	0.63	0.60	0.62	0.61	0.61	0.54	0.62	0.61
Pre_PV	0.59	0.54	0.58	0.59	0.61	0.60	0.60	0.57	0.56	0.53	0.59	0.57
TN_PP	13.00	25.00	16.00	19.00	20.00	26.00	14.00	16.00	17.00	24.00	20.00	17.00
TP_PP	52.00	31.00	55.00	49.00	49.00	41.00	46.00	51.00	56.00	33.00	42.00	45.00
FN_PP	15.00	36.00	12.00	18.00	18.00	26.00	21.00	16.00	11.00	34.00	25.00	22.00
FP_PP	20.00	8.00	17.00	14.00	13.00	7.00	19.00	17.00	16.00	9.00	13.00	16.00
Acc_PP	0.65	0.56	0.71	0.68	0.69	0.67	0.60	0.67	0.73	0.57	0.62	0.62
AUC_PP	0.68	0.67	0.70	0.67	0.71	0.70	0.65	0.70	0.72	0.65	0.65	0.64
Sen_PP	0.78	0.46	0.82	0.73	0.73	0.61	0.69	0.76	0.84	0.49	0.63	0.67
Spe_PP	0.39	0.76	0.48	0.58	0.61	0.79	0.42	0.48	0.52	0.73	0.61	0.52
BAC_PP	0.59	0.61	0.65	0.65	0.67	0.70	0.56	0.62	0.68	0.61	0.62	0.59
Pre_PP	0.72	0.79	0.76	0.78	0.79	0.85	0.71	0.75	0.78	0.79	0.76	0.74

WoAug: Without Augmentation, WAug: With Augmentation, TN: True Negative, TP: True Positive, FN: False Negative, FP: False Positive, Acc: Accuracy, AUC: Area Under ROC Curve, Sen: Sensitivity, Spe: Specificity, BAC: Balanced Accuracy, Pre: Precision, LAD: Left Anterior Descending artery, RCA: Right Coronary Artery, LCX: Left Circumflex artery, PV: Per-vessel analysis, PP: Per-patient analysis.

Table S3. The best model performance in each strategy for per patient analysis in ICA-based diagnosis.

Strategy	1-WoAug	1-WAug	2-WoAug	2-WAug	3-WoAug	3-WAug	4-WoAug	4-WAug	5-WoAug	5-WAug	6-WoAug	6-WAug
Model	CNN2	InceptionV3	InceptionResNetV2	InceptionResNetV2	InceptionResNetV2	DenseNet201	DenseNet201	ResNet50V2	ResNet152V2	VGG19	DenseNet201	ResNet50V2
Input	EarlyFusion	Stress	EarlyFusion	EarlyFusion	EarlyFusion	LateFusion	EarlyFusion	Stress	EarlyFusion	LateFusion	EarlyFusion	Stress
TN_LAD	23.00	39.00	29.00	24.00	35.00	33.00	29.00	20.00	36.00	18.00	32.00	20.00
TP_LAD	35.00	12.00	30.00	32.00	26.00	23.00	26.00	36.00	35.00	43.00	28.00	38.00
FN_LAD	22.00	45.00	27.00	25.00	31.00	34.00	31.00	21.00	22.00	14.00	29.00	19.00
FP_LAD	20.00	4.00	14.00	19.00	8.00	10.00	14.00	23.00	7.00	25.00	11.00	23.00
Acc_LAD	0.58	0.51	0.59	0.56	0.61	0.56	0.55	0.56	0.71	0.61	0.60	0.58
AUC_LAD	0.65	0.63	0.67	0.62	0.66	0.65	0.66	0.59	0.80	0.63	0.67	0.60
Sen_LAD	0.61	0.21	0.53	0.56	0.46	0.40	0.46	0.63	0.61	0.75	0.49	0.67
Spe_LAD	0.53	0.91	0.67	0.56	0.81	0.77	0.67	0.47	0.84	0.42	0.74	0.47
BAC_LAD	0.57	0.56	0.60	0.56	0.64	0.59	0.57	0.55	0.73	0.59	0.62	0.57
Pre_LAD	0.64	0.75	0.68	0.63	0.76	0.70	0.65	0.61	0.83	0.63	0.72	0.62
TN_RCA	28.00	42.00	38.00	37.00	40.00	44.00	29.00	37.00	35.00	24.00	30.00	35.00
TP_RCA	30.00	18.00	22.00	25.00	26.00	21.00	29.00	26.00	21.00	30.00	28.00	28.00
FN_RCA	9.00	21.00	17.00	14.00	13.00	18.00	10.00	13.00	18.00	9.00	11.00	11.00
FP_RCA	33.00	19.00	23.00	24.00	21.00	17.00	32.00	24.00	26.00	37.00	31.00	26.00
Acc_RCA	0.58	0.60	0.60	0.62	0.66	0.65	0.58	0.63	0.56	0.54	0.58	0.63
AUC_RCA	0.62	0.66	0.65	0.65	0.70	0.66	0.63	0.67	0.55	0.60	0.65	0.66
Sen_RCA	0.77	0.46	0.56	0.64	0.67	0.54	0.74	0.67	0.54	0.77	0.72	0.72
Spe_RCA	0.46	0.69	0.62	0.61	0.66	0.72	0.48	0.61	0.57	0.39	0.49	0.57
BAC_RCA	0.61	0.58	0.59	0.62	0.66	0.63	0.61	0.64	0.56	0.58	0.60	0.65
Pre_RCA	0.48	0.49	0.49	0.51	0.55	0.55	0.48	0.52	0.45	0.45	0.47	0.52
TN_LCX	29.00	44.00	33.00	37.00	31.00	40.00	32.00	39.00	29.00	28.00	27.00	41.00
TP_LCX	35.00	13.00	33.00	27.00	33.00	23.00	30.00	24.00	29.00	32.00	32.00	26.00
FN_LCX	8.00	30.00	10.00	16.00	10.00	20.00	13.00	19.00	14.00	11.00	11.00	17.00
FP_LCX	28.00	13.00	24.00	20.00	26.00	17.00	25.00	18.00	28.00	29.00	30.00	16.00
Acc_LCX	0.64	0.57	0.66	0.64	0.64	0.63	0.62	0.63	0.58	0.60	0.59	0.67
AUC_LCX	0.71	0.68	0.77	0.69	0.73	0.68	0.60	0.72	0.59	0.67	0.64	0.72
Sen_LCX	0.81	0.30	0.77	0.63	0.77	0.53	0.70	0.56	0.67	0.74	0.74	0.60
Spe_LCX	0.51	0.77	0.58	0.65	0.54	0.70	0.56	0.68	0.51	0.49	0.47	0.72
BAC_LCX	0.66	0.54	0.67	0.64	0.66	0.62	0.63	0.62	0.59	0.62	0.61	0.66

Pre_LCX	0.56	0.50	0.58	0.57	0.56	0.58	0.55	0.57	0.51	0.52	0.52	0.62
TN_PV	80.00	125.00	100.00	98.00	106.00	117.00	90.00	96.00	100.00	70.00	89.00	96.00
TP_PV	100.00	43.00	85.00	84.00	85.00	67.00	85.00	86.00	85.00	105.00	88.00	92.00
FN_PV	39.00	96.00	54.00	55.00	54.00	72.00	54.00	53.00	54.00	34.00	51.00	47.00
FP_PV	81.00	36.00	61.00	63.00	55.00	44.00	71.00	65.00	61.00	91.00	72.00	65.00
Acc_PV	0.60	0.56	0.62	0.61	0.64	0.61	0.58	0.61	0.62	0.58	0.59	0.63
AUC_PV	0.68	0.68	0.71	0.67	0.71	0.68	0.65	0.67	0.64	0.63	0.64	0.67
Sen_PV	0.72	0.31	0.61	0.60	0.61	0.48	0.61	0.62	0.61	0.76	0.63	0.66
Spe_PV	0.50	0.78	0.62	0.61	0.66	0.73	0.56	0.60	0.62	0.43	0.55	0.60
BAC_PV	0.61	0.54	0.62	0.61	0.63	0.60	0.59	0.61	0.62	0.60	0.59	0.63
Pre_PV	0.55	0.54	0.58	0.57	0.61	0.60	0.54	0.57	0.58	0.54	0.55	0.59
TN_PP	16.00	25.00	16.00	22.00	20.00	26.00	16.00	16.00	20.00	15.00	16.00	18.00
TP_PP	55.00	31.00	55.00	40.00	49.00	41.00	52.00	51.00	56.00	57.00	56.00	54.00
FN_PP	12.00	36.00	12.00	27.00	18.00	26.00	15.00	16.00	11.00	10.00	11.00	13.00
FP_PP	17.00	8.00	17.00	11.00	13.00	7.00	17.00	17.00	13.00	18.00	17.00	15.00
Acc_PP	0.71	0.56	0.71	0.62	0.69	0.67	0.68	0.67	0.76	0.72	0.72	0.72
AUC_PP	0.70	0.67	0.70	0.69	0.71	0.70	0.69	0.70	0.77	0.66	0.73	0.71
Sen_PP	0.82	0.46	0.82	0.60	0.73	0.61	0.78	0.76	0.84	0.85	0.84	0.81
Spe_PP	0.48	0.76	0.48	0.67	0.61	0.79	0.48	0.48	0.61	0.45	0.48	0.55
BAC_PP	0.65	0.61	0.65	0.63	0.67	0.70	0.63	0.62	0.72	0.65	0.66	0.68
Pre_PP	0.76	0.79	0.76	0.78	0.79	0.85	0.75	0.75	0.81	0.76	0.77	0.78

WoAug: Without Augmentation, WAug: With Augmentation, TN: True Negative, TP: True Positive, FN: False Negative, FP: False Positive, Acc: Accuracy, AUC: Area Under ROC Curve, Sen: Sensitivity, Spe: Specificity, BAC: Balanced Accuracy, Pre: Precision, LAD: Left Anterior Descending artery, RCA: Right Coronary Artery, LCX: Left Circumflex artery, PV: Per-vessel analysis, PP: Per-patient analysis.
